# The Relation of the Dental Profession to the Government

**Published:** 1918-11

**Authors:** 


### THE RELATION OF THE DENTAL PROFESSION TO THE GOVERNMENT

By a few men in the profession the full significance of the legislation at Washington last October was realized, but by only a few. When Congress passed a law granting to dentistry equal status with medicine in the army, a most momentous thing happened affecting the future welfare of the profession. It seems so difficult to get the rank and file of dentistry to comprehend just what this thing involves. Most of the comment on this legislation has been in the nature of congratulating ourselves on the recognition which thereby came to dentistry, but this is far from being the really significant thing in the situation. It is true that dentistry may well be congratulated on the recognition thus given it, but attention should be directed less to the honor which has come to the profession and more to the responsibility which it involves. Never before has so much been accorded to dentistry, and never before has so much been demanded of it; and it is this latter fact which seems to entirely escape the attention of many in the profession.

Woe betide dentistry if we do not measure up to the estimate which the Government has placed upon us. More and more the Government reaches out and asks us to help. It is a time of emergency and stress, and the Government has wakened to the fact that there are many ways in which we can be of service. First, it accepted the beneficent offices of the Preparedness League of American Dentists in putting the mouths of the recruits in good condition before going to the various cantonments; next, it requested that a dentist should serve on each Advisory Board in connection with the draft, and now it suggests that a dentist should also be represented in each Local Board. Be it said to the honor of many men in the profession that they have responded most loyally to these various appeals. Tens of thousands of free dental operations have been performed for the drafted men, dentists have served whole-heartedly on the Advisory Boards, and others will serve just as willingly on the Local Boards. The Dental Reserve Corps in the army is well organized, and it is making a good impression on the War Department. Everything on the surface would seem to be working satisfactorily so far as the dental profession is concerned, but—is it?

Not one-half is being done which might be done. There are probably less than 15,000 members of the Preparedness League. This would seem to be a pretty big membership but it should be at least double that number. Thirty thousand dentists banded together in one organization would mean something. (By the way, this is the mark set for membership in the National Dental Association for the 1918 meeting, and we must reach that number.) Instead of tens of thousands of free dental operations, we should have hundreds of thousands. We can not do too much for the boys who are going to fight our battles for us. Then the work on the Advisory and Local Boards should be divided up more than it is, so that a few men are not obliged to make all the sacrifice.

The disposition on the part of some men to "let others attend to it" is altogether too prevalent. This is a time when every man must do his share. There is not a man or woman practicing dentistry today who is not capable of helping in some capacity, and there should be such a generous and spontaneous response on the part of the profession when appeals are made on behalf of any of the war activities, that the Government will perforce be impressed with the fact that dentists are fully alert to the exigencies of the present situation, and are loyally and earnestly anxious to accept their full responsibility in the matter.

Do not wait to be asked and urged to do what is your bounden duty, but search out the ways and means by which you can do the greatest good, and then go wholeheartedly at it. Remember that while we are at heart a peace-loving people, the developments of the recent past have forced on us a terrible war, and we must see it through to a successful issue. There is no escaping it, even if we would. And the sooner we realize the fact that the chief business of this nation at the present time is WAR, the better it will be for us. Everything which does not contribute in some way toward winning the war is worse than wasted. At this time every bit of energy we employ for ulterior or irrelevant purposes tends to prolong the war just that much. Let this sink into our consciousness, and as a profession let us get behind the Government to a man, and prove that we are at least worthy of the confidence which the Government has reposed in us.—Editorial in June *Journal N. D. A.*

